# The Relationship Between Paternal Smoking and Overweight/Obesity with Childhood Overweight/Obesity: A Systematic Review

**DOI:** 10.1007/s13679-025-00617-z

**Published:** 2025-03-28

**Authors:** Usama Hussain, Nida Ziauddeen, Elizabeth Taylor, Nisreen A. Alwan

**Affiliations:** 1 Royal Berkshire Hospital, Reading, UK; 2 School of Primary Care, Population Sciences and Medical Education, Faculty of Medicine, University of Southampton, Southampton, UK; 3 Nuffield Department of Population Health, University of Oxford, Oxfordshire, UK; 4 University Hospital Southampton NHS Foundation Trust, Southampton, UK; 5 NIHR Applied Research Collaboration Wessex, Southampton, England

**Keywords:** Childhood obesity, Paternal obesity, Paternal smoking, Childhood overweight, Paternal overweight

## Abstract

**Purpose of Review:**

This review investigates the relationship of paternal smoking and overweight/obesity during pregnancy and up to one-year post-birth with childhood overweight/obesity up to age 12. Both exposures were analysed separately and together, if appropriate.

**Recent Findings:**

Included studies indicate that paternal overweight/obesity is consistently associated with increased risk of childhood overweight/obesity, suggesting a robust intergenerational link. Conversely, findings on paternal smoking are less consistent. Five out of six studies suggest that paternal smoking during pregnancy may contribute to increased risk but one found no association.

**Summary:**

Children of fathers with overweight/obesity are at higher risk of overweight/obesity in childhood. Paternal smoking was associated with higher risk of child overweight/obesity in most studies. Trajectories of overweight and obesity are likely to be transgenerational and systemic changes to tackle their socioeconomic determinants may be required to address these.

## Introduction

Overweight and obesity are global health issues, defined as abnormal or excessive fat accumulation that may harm health. Among children and adolescents worldwide, the rate of obesity in 2022 was four times higher than in 1990 [1]. The rise in childhood obesity has heightened focus on its long-term health consequences.

Research has primarily focused on maternal factors and childhood overweight/obesity, with less emphasis on paternal factors [2]. One review highlighted the underrepresentation of fathers in studies on parental factors and childhood obesity, with fathers making up only 17% of parent participants in 667 studies [3]. Evidence suggests that paternal health is crucial; a cross-sectional study found children of fathers with obesity are at higher risk of metabolic disease, regardless of maternal weight [4].

Paternal smoking also impacts childhood health. A review of 14 studies found children whose mothers smoked during pregnancy were at increased risk of being overweight between ages 3–33 [5]. Limited research exists on the effect of paternal smoking on childhood overweight/obesity. An individual participant data meta-analysis of 229,158 families across 28 birth cohorts showed a higher risk of childhood overweight in children whose mothers smoked during the first trimester and throughout pregnancy, compared to those whose mothers did not smoke [6]. This analysis also found that paternal smoking was associated with a higher risk of childhood overweight, independent of maternal smoking. However, the study focused on Europe and North America, and did not extend the observation period beyond 1-year post-birth, or tracking obesity risk progression over time [6].

A UK based cross-sectional study found no association between paternal smoking and childhood overweight/obesity but did find an association between maternal smoking and childhood overweight/obesity when considering prenatal cigarette smoke exposure [7]. A previous systematic review examining the association between environmental smoking exposure and childhood obesity found a positive association for both maternal and paternal smoking, with a higher effect estimate for maternal smoking [8]. However, this review focussed on environmental smoking exposure for the mother and source of exposure was unclear in some of the included studies, our review aims to focus solely on paternal smoking during the pregnancy period.

When examining the relationship between paternal smoking and child obesity, any observed association may be due to confounding factors like paternal obesity. People with overweight/obesity may be more likely to start smoking [9]. A Mendelian randomization study from the UK Biobank showed each standard deviation increment in BMI increased the risk of being a smoker [9].

We aimed to systematically review the literature to examine the consistency of evidence exploring the relationship between paternal smoking and/or paternal overweight/obesity with childhood overweight/obesity.

## Methods

### Search Strategy

The review questions were structured using the PICO framework. The population included children up to the age of 12. The intervention/exposure was paternal smoking and/or paternal overweight/obesity. The comparator was paternal non-smoking and/or father without overweight/obesity. The outcome was defined as childhood overweight or obesity based on the study criteria.

An electronic search was conducted using the bibliographic databases: EMBASE (via Ovid), MEDLINE (via Ovid), CINAHL (via EBSCO) and Web of Science. The search was run initially on 27th September 2020 and then updated on 10th May 2024.

The search strategy was developed in consultation with a specialist librarian and was piloted before finalising. The search strategy was piloted and run initially on Medline Ovid, then adapted appropriately for the other databases. The search strategy used was as follows:

[{obes*.mp. or overweight.mp. or BMI.mp. or body mass index.mp.} AND {child*.mp or (paediatric* or pediatric*).mp. or adolescen*.mp. or teen*.mp.} OR Pediatric Obesity/]

AND [fume.mp or smok*.mp. or nicotine.mp. or tobacco.mp. or exp Smoking/].

AND [(paternal or dad or father*).mp. and child*.mp.]

### Exposure Assessment

Paternal smoking and/or paternal overweight/obesity were considered as exposures of interest. Studies which assessed the exposure from pregnancy to 1-year post-birth were considered. This time was chosen so this review could look at early exposures during foetal development and early life. All measures and methods used to measure paternal overweight/obesity were considered appropriate for inclusion if recorded within this time frame. Paternal smoking was limited to cigarette use, excluding vaping and other products. Both measures could include self-reported questionnaires/surveys, or clinical assessments. Continuous and categorical measures of exposure and outcome were included, such as BMI, waist-to-height ratio, body fat percentage, cigarette consumption, and hours of smoke exposure.

### Outcome Assessment

Outcome measures following birth to age 12 were considered as defined by the included paper. Self-reported measures and results from healthcare settings were both deemed appropriate for inclusion.

### Inclusion and Exclusion Criteria

Longitudinal cohort and case-control observational studies were included. Cross-sectional, ecological and case studies were excluded. Studies from any country were included but had to be published in the English language. Studies were restricted to those based on human participants and published since 1990.

### Screening and Data Extraction

Identified records were exported to EndNote X9 [10] and the screening software Rayyan [11] was used in the screening process. A randomly selected sample of 10% of titles was independently screened by two reviewers (UH and EJT). It has been shown that if the proportion being sampled for independent review is increased, there is no decrease in discordance [12]. A 75% cut-off for concordance between the two reviewers was decided. If the agreement percentage was lower, the second reviewer would also review all the papers. Initially title screening was done, then abstract and finally full-text screening, using a 10% double screening sample. The records without agreement in the title screen were included in abstract screening. Following abstract screening, the full texts of the remaining records were accessed and reviewed by both reviewers. A modified version of the Cochrane collaboration data extraction form was used to extract study characteristics, information regarding participants with outcome and exposure assessment and assess risk of bias [13].

### Quality Assessment

Quality assessment was undertaken using the Critical appraisal skills programme (CASP) checklist [14]. This was done for all the papers by one reviewer (UH), with the second reviewer (EJT) carrying this out for a random selection of 20% of papers.

## Results

**Fig. 1:**
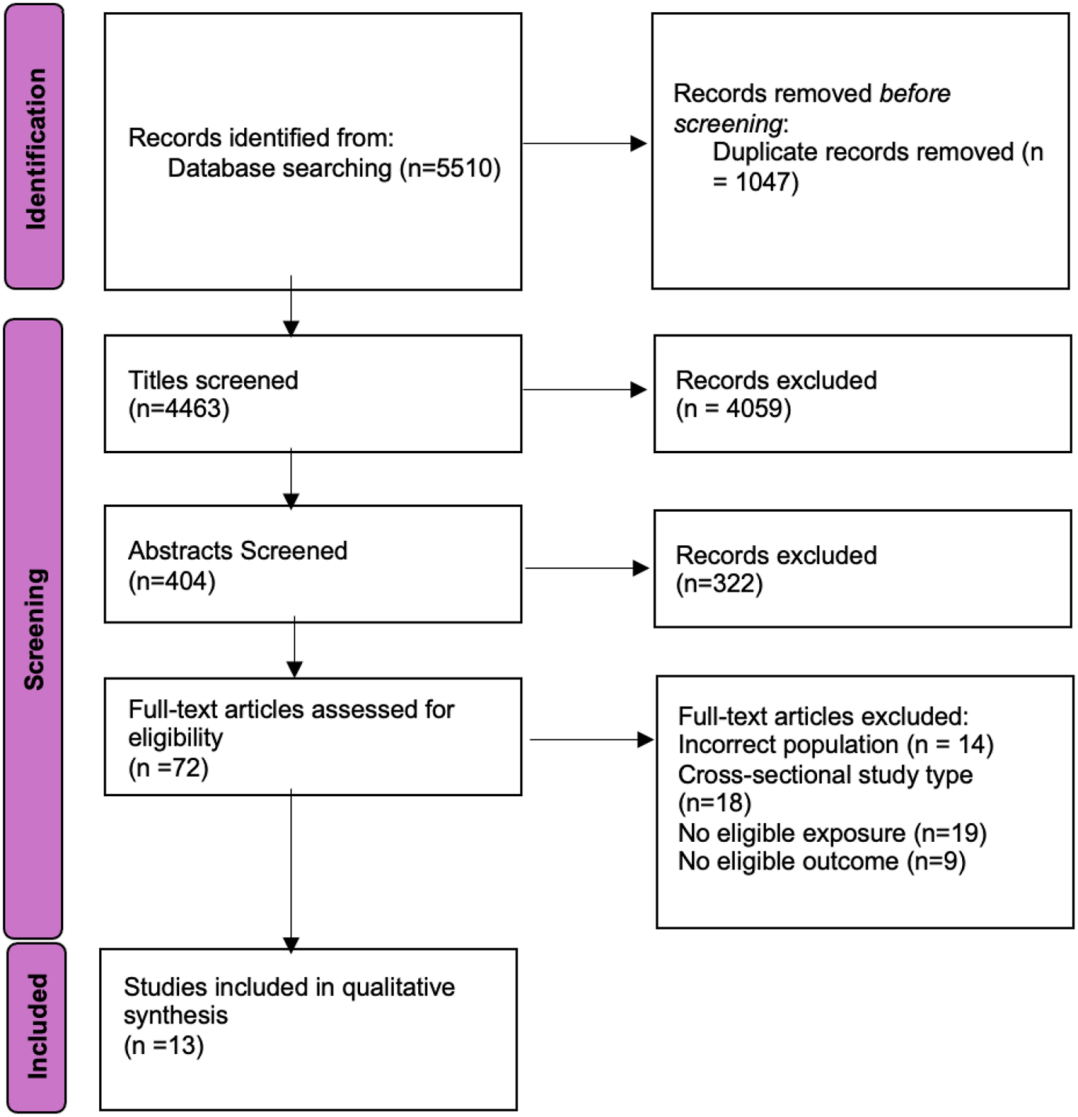
PRISMA Flow Diagram [15]

The search identified 5510 records, of which 1047 were duplicates. A total of 4463 titles were screened and 404 abstracts. The percentage agreement was 97% for title screening and 98% for abstract screening. Full texts of 72 papers were assessed and thirteen papers met the inclusion criteria (Fig. 1). Agreement was 100% for full-text screening between both reviewers. Following the completion of quality assessment, a decision was made to undertake a narrative synthesis rather than a meta-analysis due to the heterogeneity of the included papers. Specifically, the papers varied significantly in terms of their sample size and the timing of when paternal exposures were recorded. Additionally, the studies examined outcome at different time points.

Study characteristics of the included studies, including exposure and outcome measurement are presented in Table 1. Of the thirteen studies included in this systematic review, 12 were prospective cohort studies [16–19, 21–28] and one was a case-control study [20]. The sample size ranged from 509 participants [20] to 29,216 participants [22]. Six studies assessed paternal smoking [16–21], seven assessed paternal overweight/obesity [22–28], and two considered both exposures [20, 27]. The outcome of childhood overweight/obesity was assessed at different ages, from birth to 11 years. Three papers were based on the same population [16, 17, 21], the Generation R study in the Netherlands. Eight of the papers recorded the paternal exposures antenatally [16–22, 25], whilst five recorded postnatally [23, 24, 26–28]. The timing of participant recruitment in relation to birth of the child varied amongst the studies. Six papers included cohorts which recruited antenatally [16, 17, 21–23, 25] whilst the other seven recruited postnatally [18–20, 24, 26–28]. Three papers used a population from the Netherlands [16, 17, 21], two in the UK [26, 28] and one each was in Germany [18], Hong Kong [19], Iran [20], Norway [22], USA [23], China [24], Australia [25] and Sweden [27].

**Table 1:** Characteristics of included studies

First Author, Year and Country	Sample Size	Study Design	Exposure Time Point	Exposure type	Outcome Timepoint	Outcome Type
Durmus 2011 Netherlands [16]	5342	Cohort	Pregnancy 1st trimester	Paternal smoking categorised as: No, Yes - <5 cigarettes/day Yes - >4 cigarettes/day	4 years but also measured 1,2 and 3 years	BMI categories, defined as overweight (BMI 1.1–2.3 SDS) and obesity (BMI > 2.3 SDS) based on the age- and sex-adjusted BMI distributions using international criteria
Durmus 2014 Netherlands [17]	5243	Cohort	Pregnancy 1st trimester	Paternal smoking categorised as: No, Yes - <5 cigarettes per day Yes - >4 cigarettes per day.	6 years	Android: gynoid fat ratio and adiposity (DXA scan) Age- and sex-adjusted cut-off points to categorise BMI.
Florath, 2014 Germany [18]	609	Cohort	Pregnancy but recorded postnatally	Paternal smoking recorded as no or yes including number of cigarettes smoked/day.	8 years	Height/weight measured in the clinic (to calculate BMI) or self-reported if non-attendance. BMI from paediatric check-ups conducted at 1, 2, 3, 4 and 6 years of age was also used with the main time point being 8 years
Kwok, 2010 Hong Kong [19]	8327	Cohort	Pregnancy (reported by parent) but recorded at the first postnatal health clinic.	Paternal smoking recorded as the number smoked daily and number of hours of smoke exposure from lit cigarettes or exhaled by nearby smokers to the pregnant mother. Categorised as daily or occasional smoking.	7 and 11 years	Height/weight measured at health service clinics by nurses (to calculate BMI). Cut-off of 85th percentile used for overweight.
Farzaneh, 2021 Iran [20]	509	Case-control	Birth	Paternal smoking categorised as yes, or no. Paternal obesity classified as BMI > 25	6 years	Height/weight (to calculate BMI) measured in school WHO age and sex-specific growth reference charts used to categorise BMI: 245 obese and overweight children (case) with 245 normal-weight children (control)
Cajachagua-Torres, 2021 Netherlands [21]	4792	Cohort	Pregnancy 1st trimester	Paternal smoking categorised as: No, Yes - <5 cigarettes/day, Yes - >4 cigarettes/day	10 years	Android: gynoid fat ratio and adiposity (DXA scan) Age- and sex-adjusted cut-off points to categorise BMI.
Fleten, 2011 Norway [22]	29,216	Cohort	Pregnancy 2nd trimester	Paternal overweight/obesity, self-report of height and weight or from maternal report if the father had not received/ responded to questionnaire	3 years	BMI (from maternal report). Continuous values not classified into normal/ overweight/obesity.
Linabery, 2013 USA [23]	912	Cohort	1 month postnatal	Paternal overweight/obesity categorised as underweight, normal weight, overweight and obesity using self-reported BMI measurements.	3.5 years	Height/weight measured at study centre (to calculate BMI).
Mei, 2018 China [24]	2220	Cohort	Paternal weight self-reported at start of pregnancy, height measured postnatally at first month follow-up home visit.	Paternal overweight/obesity, BMI as continuous independent variable and categorised into weight status groups, based on BMI cut-offs recommended by the Working Group of Obesity in China	Outcome points from birth to 2 years, measured 4 times	Height/weight recorded by healthcare staff using measuring tape and electronic scale (to calculate BMI). Overweight and obesity defined as BMI ≥ 85th and ≥ 95th percentiles according to WHO gender-specific and age-specific values
O’Callaghan, 1997 Australia [25]	4062	Cohort	Paternal BMI during pregnancy (from maternal report)	Paternal overweight/obesity, BMI divided into percentiles for analysis: ≥94 percentile and 85th-94th percentile.	5 years	Height/weight measured (to calculate BMI) by research staff. Severe obesity categorised as BMI > 94th percentile and moderate obesity as BMI 85th -94th percentile
Weng, 2013 UK [26]	18,296	Cohort	Paternal BMI postnatally at first interview when child aged 6–12 months	Paternal overweight/obesity, reported by both parents. Categorised as underweight, normal weight, overweight and obesity according to BMI.	3 years	Defined by International Obesity Task Force (IOTF) gender and age-specific cut-off corresponding to adult BMI > 25
Lindholm, 2022 Sweden [27]	1,540	Cohort	Postnatally when infant aged 45 days	Paternal overweight/obesity with BMI as continuous values. Paternal smoking is categorised as yes, or no.	5 years	Height/weight measured by trained nurses in the clinic (to measure BMI). Sex- and age-specific scores based on Swedish reference data: overweight/obesity or normal weight/underweight
Hawkins, 2009 UK [28]	13,188	Cohort	Paternal BMI self-reported at 9 months postnatally	Categorised as: Neither parent overweight (BMI < 25 kg/m^2^) Father is overweight Mother is overweight Both parents overweight	3 years	Weight/height measured by trained interviewers (to calculate BMI): overweight (including obesity) defined using IOTF cut-offs

**Table 2:** Study findings and quality assessment

First Author, Year and Country	Main Findings	Strengths and limitations, and overall rating for risk of bias
Durmus, 2011 Netherlands [16]	Paternal smoking during pregnancy of the partner not associated with risk of overweight or obesity in the offspring at 4 years	+Smoking categorised and dose-response curve used. +Outcome measured by well-trained staff at community health centre. +BMI values adjusted for ages and sex. Adjusted for paternal BMI in the study. +Loss to follow-up less than 5%. +Analysis for paternal smoking when mother does not smoke. + Child BMI classified into overweight, or obesity based on standardised definitions. - Maternal report of paternal smoking, possible under- reporting. -Paternal smoking during pregnancy was collected without reference to postnatal growth characteristics. Rating: Fair
Durmus, 2014 Netherlands [17]	Paternal smoking during pregnancy associated with: ● higher BMI and total fat (difference: 0.09 (95% CI: 0.03–0.15) SDS) ● higher android/gynoid fat ratio (difference: 0.12 (95% CI: 0.06–0.19) SDS), increased risk of childhood overweight (OR: 1.32 (95% CI: 1.10–1.58)). No sex- specific effects (p-value for sex interaction > 0.05).	+DXA scan to measure adiposity with daily quality assurance tests, well-trained staff in dedicated research centres. +Adjusted for relevant confounders +Smoking categorised and dose-response curve used. +Examined child sex-specific effect. - Paternal smoking reported by the mother. -Only measured at one time point. -Recorded number of cigarettes smoked daily rather than the number of cigarettes a child is exposed to. -30% of participants lost to follow-up at the 6-year follow-up Rating: Good
Florath, 2014 Germany [18]	Moderate but significant association with offspring BMI when only father smoked in pregnancy. In multivariate analysis, paternal smoking during pregnancy was associated with a higher BMI (difference: 0.34 (95% CI: 0.01–0.66))	+ Outcome reported at multiple time points during the offspring’s life. + Large number of confounders accounted for considering maternal and paternal characteristics. + Maternal smoking validated using cotinine umbilical blood levels. + Analysis for paternal smoking in non-smoking mothers. -Recruited postnatally so there is a risk of bias in exposure assessment. - Excluded low birth weight children which may have reduced the association. - Follow-up rate was only 59% and those who still participated were older and of a higher socioeconomic status, risk of participation bias. Rating: Fair
Kwok, 2010 Hong Kong [19]	Paternal smoking associated with greater offspring BMI. Paternal smoking daily associated with children who are overweight/obesity at both 7 and 11 years (7yrs difference: 0.15 (95% CI: 0.003–0.29) (11yrs: 0.14 (95% CI: 0.04–0.25). No statistically significant difference in BMI with occasional paternal smoking at both ages (7yrs difference: 0.1 (95% CI: − 0.12–0.32)), (11 yrs difference: 0.11 (-0.03 to 0.25))	+Maternal smokers excluded in analysis of paternal smoking + Fathers who smoke measured as the number of cigarettes smoked and hours daily mother exposed to paternal smoking. +Analysis for paternal smoking when mother does not smoke. +Child BMI categorised into overweight/obesity. - Recruited postnatally, risk of bias in paternal exposure. - Follow-up rate only 82%. - Overweight/obesity in mothers and fathers not adjusted for; results may be biased. Rating: Poor
Farzaneh, 2021 Iran [20]	Significant association between paternal smoking and risk of paediatric obesity. In multivariate regression analysis, paternal smoking associated with increased risk of childhood obesity, (OR) 1.84 (95% CI: 1.15, 2.94; *p* = 0.001). Paternal BMI is excluded from stepwise logistic regression, due to multiple linear correlations. 25.6% of children with overweight/obesity had fathers with overweight/obesity compared to 14.7% in the children of normal weight.	+Outcome measured using standardised clinical methods +The study included a considerable number of participants, which strengthens validity of findings -The study used data from routine healthcare records, which may not represent all populations, leading to potential selection bias. - Study seems to report on all measured outcomes, but the exclusion of paternal BMI from the final model might indicate selective reporting. Rating: Poor
Cajachagua-Torres, 2021 Netherlands [21]	Paternal smoking during pregnancy was associated with higher risk of overweight and obesity in children at 10 years. Those exposed to paternal smoking during pregnancy had a higher BMI (difference 0.13 SDS, 95% CI 0.06–0.19). Higher android/gynoid fat mass ratio (difference 0.17 SDS, 95% CI 0.11–0.23), higher fat mass index (difference 0.20 SDS, 95% CI 0.12–0.28) and higher risk of overweight (OR 1.30, 95% CI 1.09–1.55). Dose–response association with highest effect estimates in children whose fathers smoked ≥ 5 cigarettes per day	+ The study minimised selection bias by using a population-based cohort. + Large sample size and prospective nature of the study help mitigate this risk - Use of questionnaires to assess parental substance use might introduce recall bias. - While the study adjusted for many confounders, residual confounding by unmeasured factors cannot be entirely ruled out. - Maternal characteristics not adjusted for Rating: Fair
Fleten, 2011 Norway [22]	Modest positive association of paternal-offspring BMI. In crude regression analyses, the paternal-offspring BMI correlation is 0.097 (95% CI: 0.086, 0.108). 1-kg/m^2^ increase in paternal BMI was associated with a 0.05-kg/m^2^ increase in offspring BMI (95% CI: 0.040, 0.051)	+ Large cohort size and prospective design. + 95% of the trios in the study had a shared obesogenic environment. + Adjusted for maternal BMI. - Paternal BMI is not classified e.g., overweight, obese. - Outcome self-reported by the mother was not validated. - Exposure measures are not consistent, some recorded by the mother and some by the father. - Equipment used to measure the outcome and exposure was not standardised, non-differential information bias. Rating: Poor
Linabery, 2013 USA [23]	Paternal BMI category was associated with infant BMI growth in the independent model (*P* = 0.01). Difference observed between infants of fathers with obesity than fathers who are overweight (*P* = 0.03) or of healthy weight (*P* = 0.005).	+ Recruited antenatally and so prospective. + Classified paternal BMI into groups. + Growth curve modelling in all infants to measure individual-level change. - Cohort mainly middle-class European Americans, homogenous population. - Infant BMI not classified as overweight or obese. - Not all exposure measures were measured prenatally, measured at different time points. -Long duration of study (1928–2012), reliability issue. Rating: Fair
Mei, 2018 China [24]	Paternal overweight/obesity was significantly associated with children’s BMI z-scores after birth but not with BMI z-scores at birth (at birth: β = 0.04, *P* > 0.05); after birth: β = 0.04– 0.20, *P* < 0.05)	+ Outcome measures in all participants measured within 10 days of the exact month. + Longitudinal + Classified paternal and child BMI into groups using WHO reference standards. - Recruited post-birth so risk of bias for exposure measures. - Parental weight and height measured at different time points. Changes in weight not captured. - Did not control for paternal smoking, only second-hand smoking. - Short follow-up duration. Low follow-up rate of 54%. Rating: Fair
O’Callaghan, 1997 Australia [25]	Paternal overweight associated with both moderate and severe childhood overweight/obesity. In the adjusted model, fathers with moderate obesity associated with moderate (OR: 1.0, 95% CI: 0.6–1.5) and severe childhood obesity (OR: 2.8, 95% CI: 1.8–4.5). Fathers with severe obesity associated with moderate (OR: 2.1, 95% CI: 1.4–3.3) and severe childhood obesity (OR: 2.0, 95% CI: 1.1–3.6).	+Recruited antenatally so prospective. Large cohort. +Classified child BMI into obese or not. +Prediction model used to establish the risk factors for obesity in children. − 35% lost to follow-up, those who were lost were more socially disadvantaged. - Paternal BMI recorded from maternal reports, no record on when data was collected. - Weighing scale for measuring the outcome is only accurate to 0.2 kg. -Obesity/overweight classification based on centiles in cohort rather than an external standard. - Did not adjust for smoking history. Rating: Fair
Weng, 2013 UK [26]	Paternal overweight/obesity positively associated with childhood overweight: OR:1.57 (95% CI: 0.79–3.10) for paternal overweight and OR:1.98 (95% CI: 1.00-3.96) for paternal obesity. Paternal BMI is one of the strongest risk factors in the prediction model for overweight in children.	+ Paternal BMI split into subgroups. + Used international guidelines for classification of exposure and outcome. - Recruited postnatally and so risk of bias in exposure measures. Risk algorithm was created, accuracy issues. - Did not adjust for paternal smoking. Indirect effects and path structure of the prediction variables was not determined. - Unknown whether the model created can predict longer term outcomes due to short follow-up length. - Overrepresentation of deprived population in this study with 60% of children from households that earned £20,800 or less, study may not be generalizable. Rating: Fair
Lindholm, 2022 Sweden [27]	Paternal BMI significantly associated with an increased waist-to-height ratio (WHtRSDS) at 5 years. Univariable logistic regression had OR 1.09 (95% CI: 1.02–1.16, *p* = 0.007) for paternal BMI and OR 0.20 (95% CI: 0.85–2.14, *p* = 0.202) for paternal smoking In multivariable logistic regression, only paternal BMI was considered-the association remained significant with an OR of 1.11 (95% CI: 1.01–1.21, *p* = 0.028)	+ The study included a population-based sample of 1,540 children, which reduces the risk of selection bias. + Outcomes like WHtRSDS and BMI were measured using standardised methods, which reduces detection bias. + The study made significant efforts to adjust for confounders, which strengthens the validity of the findings. - Response rate and criteria for inclusion not stated. - The study relied on parental reports for some data, such as BMI and feeding practices, which could introduce reporting bias. - No information about dropout rate or how missing data were handled, which is necessary to assess attrition bias. Rating: Fair
Hawkins, 2009 UK [28]	In the unadjusted model, paternal overweight associated with childhood obesity (OR: 1.78, 95% CI: 1.54–2.05, *p* < 0.001). In the adjusted model, the association for paternal overweight was lower (OR: 1.45, 95% CI: 1.28–1.63, *p* < 0.05). When both parents were overweight, the association strengthened (OR: 1.89, 95% CI: 1.63–2.19).	+ Study employs a thorough approach to adjust for confounding variables, using a stepped analysis to examine the impact of sequential adjustment on risk factors. This method helps in understanding the causal pathways and identifying modifiable risk factors + Estimates of parental overweight in the study are similar to those reported in national surveys, suggesting reasonable accuracy. - The study relies on self-reported height and weight for parents, which can lead to underestimation of obesity - Information on health behaviours during pregnancy and early postpartum was collected retrospectively, risk of recall bias Rating: Fair

**Table 3:** Summary of findings ( + = positive association) and confounders adjusted for in the included studies

First author, year and country	Paternal overweight/obesity	Paternal smoking	Variables adjusted for			
			Maternal factors	Paternal factors	Child factors	Other
Durmus, 2011 Netherlands [16]	N/A	no association	• Ethnicity • Education • Height and weight • Breastfeeding (yes/no)	• Ethnicity • Education • Height and weight • Number of cigarettes smoked by the father during pregnancy	• Age at visit (second, third trimester; birth; 3, 6, 12, 24, 36, or 48 months) • Sex	Postnatal smoking, parity, and maternal alcohol consumption excluded as did not significantly affect the estimates.
Durmus, 2014 Netherlands [17]	N/A	+	• Ethnicity • Education • BMI at enrolment • Parity • Age • Folic acid use • Gestational weight gain • Alcohol consumption • Breastfeeding	• Ethnicity • Education • BMI at enrolment	• TV watching • Age at visit • Sex • Height	Paternal smoking analyses limited to children of mothers who did not smoke during pregnancy
Florath, 2014 Germany [18]	N/A	+	• Pre-pregnancy BMI • Breastfeeding	• BMI • Education	• Gender • Gestational age at birth • Height at 8 years • BMI at birth • Monthly weight gain from birth to 1 year of age • TV watching • Sports activities • Diet score at 8 years • Age at anthropometric measurements	-
Kwok, 2010 Hong Kong [19]	N/A	+	• Highest parental education • Highest parental occupation • Place of birth • Breastfeeding history • Household income per person	• Highest parental education • Highest parental occupation • Household income per person	• Sex • Birth order • Pubertal status (for analysis at 11 years) • Hospitalizations for treatment of infections (0–6 months)	Considered whether associations varied by gender based on interaction terms.
Farzaneh, 2021 Iran [20]	N/A	+	• Pre-pregnancy BMI • High gestational weight gain • Gestational diabetes • Smoking during pregnancy	-	• Birthweight > 4000 g • Time of solid food introduction (< 4 months)	Child’s physical activity and sleep rate, and paternal BMI considered but unclear if adjusted for in final analysis.
Cajachagua-Torres, 2021 Netherlands [21]	N/A	+	• Age • Education • Ethnicity • Alcohol use • Pre-pregnancy BMI • Psychopathology score (measured using Brief Symptom Inventory (BSI))	-	• Sex • Age	Foetal exposure to tobacco and cannabis (paternal and maternal smoking and cannabis use) considered. Interaction terms were tested for parental smoking/cannabis use with child sex and maternal ethnicity
Fleten, 2011 Norway [22]	+	N/A	• Coffee consumption during pregnancy • Pre-pregnancy BMI • Smoking during pregnancy • Education • Gestational diabetes • Diet during pregnancy • Gestational weight change • Breastfeeding • Postnatal smoking	• Education • Smoking during pregnancy and/or at offspring age 18 months	• Outdoor activities • Watching television/video • Diet • Day care	Number of siblings
Linabery, 2013 USA [23]	+	N/A	• BMI • Age • Smoking • Stature	• Age • Smoking • Stature	• Sex • Gestational age at birth • Birth order • Birth year tertile • Age	-
Mei, 2018 China [24]	+	N/A	• Age • Education • Gestational weight gain • Mode of birth	-	• Sex • Gestational age at birth	-
O’Callaghan, 1997 Australia [25]	+	N/A	• Pre-pregnancy BMI • Education • Income	-	-	-
Weng, 2013 UK [26]	+	N/A	• Smoking during pregnancy • Breastfeeding • Pre-pregnancy BMI	-	• Sex • Birthweight • Rapid weight gain in first year	-
Lindholm, 2022 Sweden [27]	+	N/A	• Pre-pregnancy BMI	-	• Rapid weight gain (0–6 and 6–12 months) • Milk cereal drink at 24 months • Bottle feeding at 12 and 24 months	-
Hawkins, 2009 UK [28]	+	N/A	• Smoking during pregnancy • Pre-pregnancy overweight • Employment • Lone motherhood status	-	• Birthweight (adjusted for gestational age and sex) • Sex • Ethnicity • Duration of breastfeeding • Introduction of solid foods • TV viewing	• Access to a garden • UK country • Ward type (area factor)

### Paternal Overweight/Obesity and Childhood Overweight/Obesity

All studies [20, 22–28] found a positive association between paternal overweight/obesity and childhood overweight/obesity, with paternal BMI used as the measure. Two studies [22, 23] adjusted for paternal smoking, while five did not [24–28]. Seven studies adjusted for maternal BMI [22–28]. One study, where the main exposure was paternal smoking, did not consider paternal BMI in multivariate analysis due to multiple linear correlations with high weight at birth and its prominent impact [20]. Two studies recorded fathers with overweight/obesity antenatally [22, 25] whilst five recorded postnatally [23, 24, 26–28], all within the 1-year inclusion period. Three studies measured overweight/obesity in children at multiple time points but found no significant association beyond early childhood [23, 24, 27]. None examined gender-based subgroup analysis.

Quality assessments of each paper can be found in Table 2. Six studies were rated as fair and one as poor. The study that was assessed as poor relied on maternal self-report of child BMI which could cause non-differential information bias in outcome measurement [22]. One study measured paternal exposure using weight and height values recorded at different times, height at the first month home visit post-birth and weight during pregnancy. This discrepancy in measurement times means that any changes in weight during this period was not captured [24]. The risk of selection bias was increased in one study due to not all children being clinically assessed due to funding issues towards the latter part of the study follow-up and thus being excluded from the analysis [25].

Additionally, one study excluded participants with missing data, measurements outside the specified age ranges, and preterm births; excluded children had lower birth weights than those included (3495 g versus 3580 g), introducing possible bias [27]. This study only included factors with significant associations (*p* < 0.05) in the initial univariable analysis in the subsequent multivariable models [27]. One study employed a stratified sampling design to over-represent children living in disadvantaged areas and from ethnic minority groups improving generalizability of the study [28].

### Paternal Smoking and Childhood Overweight and Obesity

Five out of six papers found an association between paternal smoking during pregnancy and the first year of life with childhood overweight/obesity [17–21]. However, the findings from two papers may be biased due to lack of adjustment for relevant confounding factors [19, 20]. One study [19] did not adjust for paternal and maternal overweight/obesity whilst the other did not adjust for paternal obesity [20]; despite both finding an association in univariate regression analysis hence both have been quality assessed as poor [19, 20].

Five papers measured paternal smoking in pregnancy using questionnaires completed by the mother [16–19, 21], with one paper not specifying which parent provided the information [20]. Three papers [16, 17, 21] had the same authors from the same research group and used data from the same cohort [16, 17, 21] with the same exposure measures but at different outcome time-points (child aged 4 years [16], 6 years [17] and 10 years [21]) and using different measures. One paper [17] considered child adiposity and overweight at 6-years with a DXA scan and android: gynoid fat ratio and the other [16] considered childhood overweight/obesity at four time-points using BMI. Fathers who smoked during pregnancy in both papers were based on maternal self-report [16, 17]. Paternal smoking was completed by fathers for a subset of participants and the inter-rater agreement between the questionnaires filled by the mother and father was good (Cohen’s κ = 0.86) [16, 17]. Both papers recorded smoking as the number of cigarettes smoked daily, however the number smoked daily may differ to the number smoked in the presence of the foetus. Only one paper considered a possible child sex-specific effect, finding no association [17].

One paper was assessed to be of good quality [17], three as fair [16, 18, 21] and two as poor [19, 20]. Durmus et al. which was assessed to be of good quality adjusted for mothers who smoke, parental BMI and used different outcome measures increasing the validity of results [17]. One other paper [20] adjusted for mothers who smoke; the other studies excluded maternal smokers from the analysis [16, 18, 19, 21]. Exclusion of mothers who smoke from the analysis could decrease reliability and does not take into consideration maternal passive smoking.

The included studies were conducted in various countries across the world increasing the generalisability of the findings. One paper also considered smoking exposure from other household smokers but there may have been issues of misclassification when participants answered the questionnaires [19]. Additionally, participants were recruited postnatally so exposure was captured retrospectively. The other study rated as poor was a case-control study which grouped both overweight and obese children together. Mothers who smoke and BMI were amongst the exposures adjusted for in analysis [20]. The exposure was only considered at time of offspring birth as smoker/non-smoker.

## Discussion

### Conclusion

All included studies examining paternal overweight/obesity found that paternal overweight/obesity during pregnancy and the first year of their child’s life was associated with childhood overweight/obesity up to age 12. Five out of six papers reported a positive association between paternal smoking and child overweight/obesity with one paper finding no association with child BMI at age 4 years [16]. However, this paper used the same population as another study that found a positive association at a later follow-up (age 6 years) [17].

The extent to which the association between paternal smoking and child overweight/obesity is modified by paternal overweight/obesity is unclear, despite it being considered as a co-variable it was not considered as an effect modifier.

Five of the six studies reported a positive association between paternal smoking and childhood overweight/obesity [17–21]. However, three studies had a high risk of bias due to a lack of appropriate adjustment for confounders [18–20]. Two papers adjusted for maternal smoking [17, 20] whilst three excluded maternal smokers from the analysis [16, 18, 19]. Two studies did not adjust for parental overweight/obesity [19, 20] whilst three papers did [16–18, 21]. All the studies had a proportion of participants lost to follow-up with follow-up rates ranging from 53% [24] to 79% [16], which was dealt with using multiple imputation [18, 19, 23, 24] or excluding participants [16, 17, 21, 22, 25–28]. While exclusions are sometimes necessary to maintain data quality and consistency, they can introduce bias depending on the reasons for exclusion. For example, if the participants lost to follow-up differed systematically from those who remained.

### Assessing Exposure and Outcome Measures

Twelve papers used height and weight to calculate BMI for the outcome measure of childhood overweight/obesity. BMI is one of the simplest and easiest ways to quantify weight status but it does not account for differences in body composition, such as muscle versus fat. This is unlikely to be an issue in children as they are still growing, but all studies examining paternal overweight/obesity also used BMI. Vanderwall et al. [29] conducted an observational study into whether BMI could predict total fat mass (TFM) and percent body fat (%FAT) in a sample of children with overweight and obesity. It found that, in children younger than 9 years, BMI is only a weak to moderate predictor for both TFM and %FAT [29].

Three papers did not use centiles [19, 22, 23] whilst the other papers did [16–18, 20, 21, 24–28]. This is important for generalisability of findings. All the papers that used centiles used appropriate reference standards to classify overweight/obesity with the exception of one which classified based on the cohort [25]. Other measures of body fat include DXA and imaging techniques which measure the amount and distribution of adipose tissue used in two studies [17, 21]. Compared with BMI, central obesity measures - including waist circumference, waist: hip ratio, and waist: height ratio - are better at predicting visceral adiposity, cardiometabolic disease, and mortality [30]. One study [28] considered both BMI and waist: height ratio strengthening validity of results with strong association between paternal BMI seen across both outcomes.

### Comparison with Other Studies and Interpretation of Results

Two studies [23, 24] suggest that the association between paternal overweight/obesity and childhood overweight/obesity strengthens as the children get older. This is supported by the UK National Health Service data from 2019 which found that 8% of children with fathers who neither had overweight nor obesity had obesity, compared to 23% of children whose fathers also had overweight or obesity [31]. A Swedish cohort study [32] of 231 children showed that the link between the severity of obesity in children and parental BMI became stronger between the ages of 7 and 15 years. Chen et al. [33] found a positive association between paternal smoking and child overweight/obesity. However, socioeconomic factors likely play a significant role in these associations, with families from lower socioeconomic backgrounds facing higher rates of obesity and smoking due to factors like limited access to healthy food, lower health literacy, and greater exposure to obesogenic environments [34]. These socioeconomic inequalities likely shape both paternal behaviours and childhood outcomes, suggesting that interventions targeting childhood obesity need to address broader social determinants as well as individual behaviours.

### Strengths and Limitations

To our knowledge, this is the first time that the exposures and outcomes on this topic have been systematically reviewed based on the inclusion criteria we have used. Reviews which have considered similar exposures and outcomes differ in timepoints they included.

Strengths of this review include the comprehensive searching of four electronic databases. The search strategy used was reviewed and informed by a specialist librarian. During the selection process a second reviewer screened a randomised 10% of the papers at each stage, reducing selection bias. A pilot search was also conducted initially to help develop an understanding of the current literature. With the exception of one case-control study, all included studies were prospective cohort studies and thus had longitudinal temporal measurements. Participants were only included if fathers and their children had lived in a shared environment throughout childhood. This was done to account for both the environmental and genetic contribution of the exposure on the outcome.

Limitations include that only studies published in English were considered for inclusion thus potentially excluding relevant studies published in other languages. Due to the time pressure and limited resources, only published papers were considered. Excluding grey literature or unpublished papers introduces the risk of publication bias as studies with positive results are more represented in literature than studies with negative results [35]. We were unable to comment on whether there was a sex-specific association between the paternal exposures and child overweight/obesity as only one study considered this [17].

### Implications for Practice and Future Research

This review found a consistent association between paternal overweight/obesity during pregnancy and the first year of life with child overweight/obesity. The association between paternal smoking and child overweight/obesity was less consistent. Lifecourse trajectories of overweight and obesity are likely to be transgenerational and addressing them requires systemic changes to tackle their socioeconomic determinants.

## Data Availability

No datasets were generated or analysed during the current study.
